# Brain Differences Associated with Self-Injurious Thoughts and Behaviors: A Meta-Analysis of Neuroimaging Studies

**DOI:** 10.1038/s41598-020-59490-6

**Published:** 2020-02-12

**Authors:** Xieyining Huang, Kelly Rootes-Murdy, Diana M. Bastidas, Derek E. Nee, Joseph C. Franklin

**Affiliations:** 1 Department of Psychology, Florida State University, Tallahassee, Florida USA; 2 Department of Psychology, Georgia State University, Atlanta, Georgia USA

**Keywords:** Biomarkers, Risk factors

## Abstract

This meta-analysis aims to evaluate whether the extant literature justifies any definitive conclusions about whether and how SITBs may be associated with brain differences. A total of 77 papers (N = 4,903) published through January 1, 2019 that compared individuals with and without SITBs were included, resulting in 882 coordinates. A pooled meta-analysis assessing for general risk for SITBs indicated a lack of convergence on structural differences. When all types of control groups were considered, functional differences in the left posterior cingulate cortex (PCC), right amygdala, left hippocampus, and right thalamus were significant using multi-level kernel density analysis (*p*_corrected_ < 0.05) but nonsignificant using activation-likelihood estimation. These results suggest that a propensity for internally-oriented, emotional processing coupled with under-active pain processing could potentially underlie SITBs, but additional research is needed to test this possibility. Separate analyses for types of SITBs suggested that the brain differences associated with deliberate self-harm were consistent with the overall findings. Checkered moderator effects were detected. Overall, the meta-analytic evidence was not robust. More studies are needed to reach definitive conclusions about whether SITBs are associated with brain differences.

## Introduction

Self-injurious thoughts and behaviors (SITBs) are major public health concerns^1–3^. Researchers have proposed that SITBs result in part from biological causes in brain systems, and many studies have linked SITBs to specific brain differences^4^. Multiple researchers have proposed that examining the “suicidal brain” will improve our understanding of SITBs, accelerate the discovery of useful biomarkers, improve the identification of high-risk individuals, and inform therapeutic and pharmacological targets for treatment^5–8^. However, the results of neuroimaging studies examining SITBs vary considerably, making it difficult to synthesize evidence on specific brain differences based on qualitative reviews. As such, this study aims to quantitatively summarize existing brain imaging research on SITBs.

Among individuals who experience SITBs, recent neuroimaging studies have found differences in multiple brain regions associated with psychological traits that confer risk for SITBs. For instance, structural alterations have been reported in the ventrolateral prefrontal cortex, dorsal prefrontal cortex, and orbitofrontal cortex among suicide attempters, and these differences were considered to reflect deficient cognitive control and impaired decision-making in the context of intense emotions^9^. Other studies reported similar findings and conclusions with regard to the amygdala^10^, the anterior cingulate cortex^11^, and the midbrain/pons^12^, and their associations with traits such as impulsivity, heightened stress reactivity, emotion dysregulation, and pain perception. Given these findings, researchers have proposed that future studies should focus on examining imaging endophenotypes associated with vulnerability to SITBs^13^. Some researchers further encouraged efforts to identify a biosignature of suicide and develop a biologically-based model of suicide risk^14,15^.

Despite evidence linking brain differences with vulnerability to SITBs, neuroimaging studies often yield different results. Most brain imaging studies that examine differences between SITB and non-SITB populations detect some kind of differences (though some studies have reported null effects^16,17^), but the same findings are rarely detected across multiple studies. For example, multiple studies have found decreased gray matter volumes in suicide attempters, but the location of these differences has varied. One study found this alteration in the left superior temporal lobe and left orbitofrontal cortex^18^, another study solely discovered this difference in the insula and posterior cingulate regions^19^, and a third study found broader altered regions including the dorsolateral prefrontal cortex, orbitofrontal cortex, and the parieto-occipital cortex^20^. Given these inconsistent findings, does the existing literature justify any definitive conclusions about differences in brain structure or function among people with a history of SITBs?

According to multiple qualitative reviews of portions of this literature, the literature does justify such conclusions. Notably, however, different reviews reach different conclusions. For instance, reviews have concluded, based on heterogeneous mixes of approximately 10–20 studies each, that SITBs are associated with differences in the ventrolateral, orbital, dorsomedial, dorsolateral, and ventromedial prefrontal cortices, the anterior cingulate gyrus, the amygdala, white matter connection, and gray matter volume^5,8,21–23^. These reviews discussed studies examining a wide range of SITBs (e.g., suicide ideation, intent, lethality, NSSI) and included diverse imaging methods (e.g., CT, MRI, SPECT, PET, fMRI, DTI) and neuropsychological tasks (e.g., viewing emotional faces, Iowa Gambling Task, verbal fluency task, Stroop task). The samples included in these reviews were also diverse, with psychiatric diagnoses of Schizophrenia, Borderline Personality Disorder, Bipolar Disorder, Major Depressive Disorder, and various affective disorders. The inconsistent conclusions across these reviews leaves open many questions about potential brain structure and functional differences among people with a history of SITBs.

A quantitative meta-analytic review may help to resolve some of these questions. With qualitative methods, it is difficult to accurately weight findings and there is often a tendency to overemphasize positive findings^24^. Quantitative meta-analysis has the advantages of increasing precision in estimating effects, boosting power by combining samples, and evaluating the effects of moderators^25^. Compared to narrative reviews, however, fewer meta-analyses exist on this topic. Among the efforts, Jollant and colleagues^26^ summarized 12 structural imaging studies and found a lack of significant differences between suicide attempters and non-attempters, whereas van Heeringen and colleagues^27^ concluded that suicide attempters have reduced volumes of the rectal gyrus, superior temporal gyrus and caudate nucleus, and increased reactivity of the anterior and posterior cingulate cortices after synthesizing 6 structural and 6 functional imaging studies. It is possible that the small number of studies per analysis has precluded researchers from drawing meaningful conclusions^28^. Hence, a more comprehensive meta-analysis is needed.

The primary goal of the present study was to conduct a more comprehensive meta-analytic review of the SITB brain imaging literature. Accordingly, the meta-analysis included 77 papers, an improvement over previous reviews. This analysis will help to determine whether the extant literature justifies any definitive conclusions about brain structure or function differences among people with a history of SITBs. This is important for three reasons. First, as reviewed above, there are many inconsistent findings in the literature. Second, the existing meta-analyses were largely underpowered. Guidelines suggest that at least 20 experiments are needed to reliably detect moderate effect sizes in Activation Likelihood Estimation (ALE), a common method for performing coordinate-based neuroimaging meta-analyses^28^. Without sufficient power, true positive findings might be observed in different brain regions even for identically performed studies^29^. Therefore, a meta-analysis with a broader scope and larger power is needed to overcome the limitations and reveal a reliable pattern in the literature. Third, recent comprehensive meta-analyses in other domains have surprisingly found that the existing literature does not justify definitive conclusions about brain imaging differences. For example, across 57 studies, a meta-analysis^30^ found no evidence for brain imaging differences among depressed individuals. These findings did not prove that there are no neural correlates of depression; rather, they showed that the extant literature did not yet justify any definitive conclusions about the neural correlates of depression and clarified directions for future research aimed at investigating these correlates. Given the inconsistency of findings from the SITB brain imaging literature, the present meta-analyses may obtain similar findings and serve a similar function.

The present study represents one of the largest efforts to synthesize the evidence on structural and functional brain differences associated with SITBs. We had three major aims. First, a pooled analysis on studies examining the neural correlates of any type of SITBs was conducted to test whether certain brain changes were associated with general vulnerabilities to SITBs. Second, separate meta-analyses were conducted for each type of SITBs to examine whether a unique brain difference exists for different types of SITBs. Third, analyses were conducted to test whether differences in study designs might moderate the findings. The results of this meta-analysis will help to summarize knowledge about the association between SITBs and brain structures and functions, and may serve as a foundation for future work in this area.

## Results

### Descriptive statistics

Most of the contrasts were yielded from studies using fMRI (57.82%), followed by structural MRI (22.00%), SPECT (11.00%), DTI (6.69%), and PET (2.49%). Regarding types of SITBs, 46.49% of the contrasts examined suicide attempt, with the rest studying self-harm regardless of suicidal intent (i.e., deliberate self-harm; 14.29%), suicide ideation and plan (13.15%), NSSI (11.34%), suicide death (9.18%), all suicidal thoughts and behaviors (5.33%), and suicide risk (0.23%). More than half of the contrasts (59.64%) adopted task-based paradigms (affective tasks: 26.98%; cognitive tasks: 24.60%; pain administration: 5.67%, and other [e.g., motor tasks]: 2.38%). Regarding control group type, about half of the contrasts (52.27%) used psychiatric controls, with the rest using healthy controls (40.25%) and self-injurious controls (7.48%; e.g., suicide attempters compared with suicide ideators).

Among the 77 papers included in this meta-analysis, the sample size ranged from 18 to 272, with 48 as the median (*M* = 63.68, *SD* = 40.80). Majority of the samples (70.13%) were adult participants, followed by mixed adult and adolescent (15.58%), child or adolescent (11.69%), mixed adults and elders (1.30%). One study only included elderly participants (1.30%). The average sample age ranged from 13.51 to 79.43, with 29.81 years old as the mean (*SD* = 11.62). Half of the samples (50.65%) included at least some participants with psychiatric medication at the time of the study, with 27.27% of the samples only including unmedicated participants, and 10.38% of the samples only including medicated participants. The rest of the studies (11.69%) did not mention participants’ medication status. Regarding psychiatric diagnoses, about half of the studies (44.16%) required participants to meet criteria for Major Depressive Disorder (MDD), followed by no specific psychiatric diagnoses required (11.69%), Schizophrenia and other psychotic disorders (11.69%), mood disorders (12.99%), Borderline Personality Disorder (7.79%), other and/or multiple diagnoses (6.49%; e.g., MDD, Posttraumatic Stress Disorder, or Traumatic Brain Injury), and Bipolar Disorder (5.19%).

### Meta-analytic results

#### Overall meta-analyses

**Figure 1:**
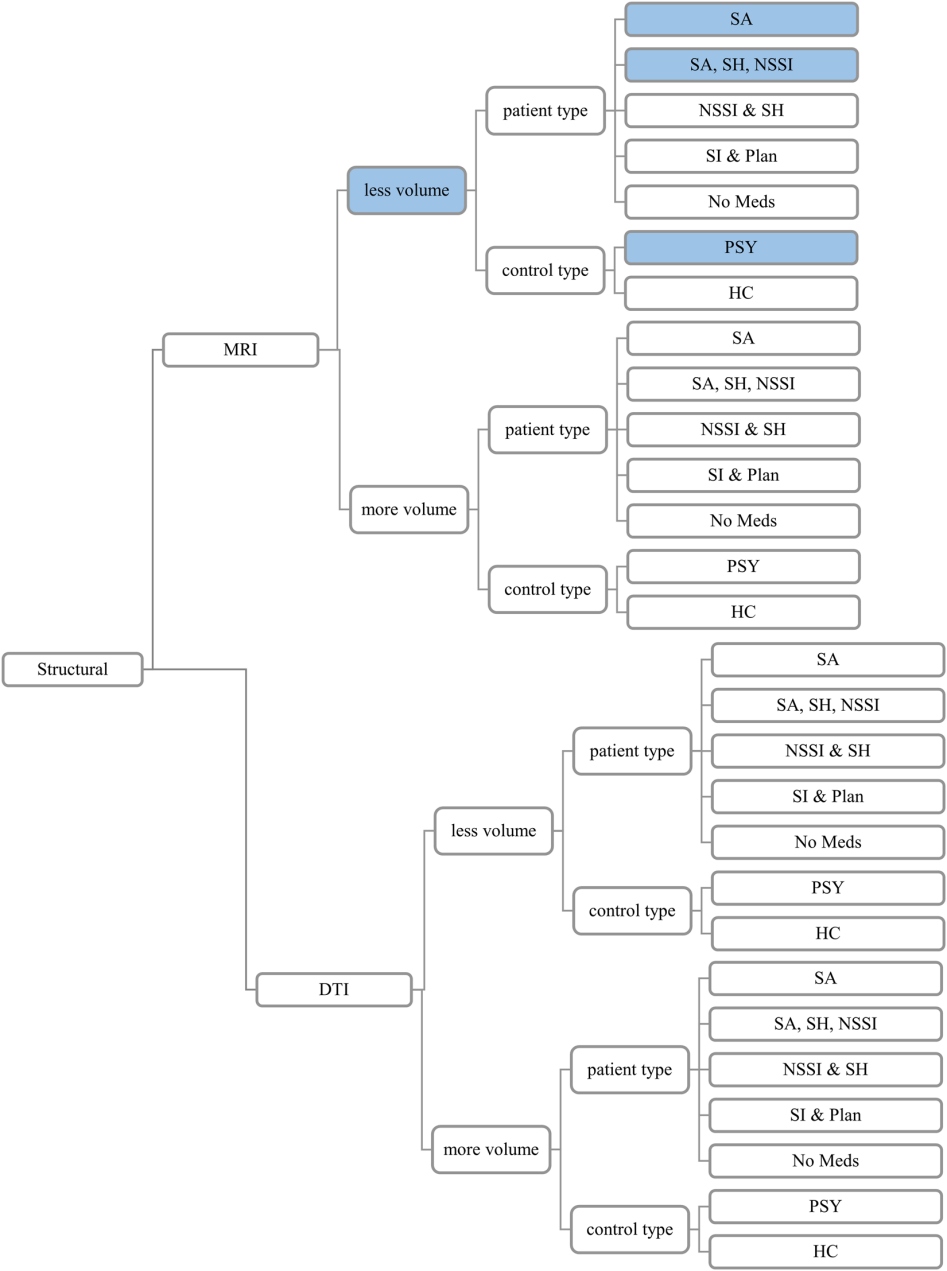
Breakdown of Structural Studies. Note. Contrasts with 10 or more experiments are highlighted in blue. There were no structural contrasts with 20 or more experiments. There were also no significant findings from the structural contrasts in either the MKDA or ALE analyses. HC: healthy controls; PSY: matched psychiatric populations; SITB: all self-injurious thoughts and behaviors; SA: suicide attempts; SH: self-harm regardless of intent; NSSI: non-suicidal self-injury; SI: suicidal ideation; DTI: diffusion tension imaging; MRI: magnetic resonance imaging.

***Structural Imaging Studies***. No structural analysis featured 20 or more experiments (Fig. 1). Although more than 10 experiments reported reduced gray matter volumes in SITBs, neither ALE nor MKDA observed a consistently significant result.

**Figure 2:**
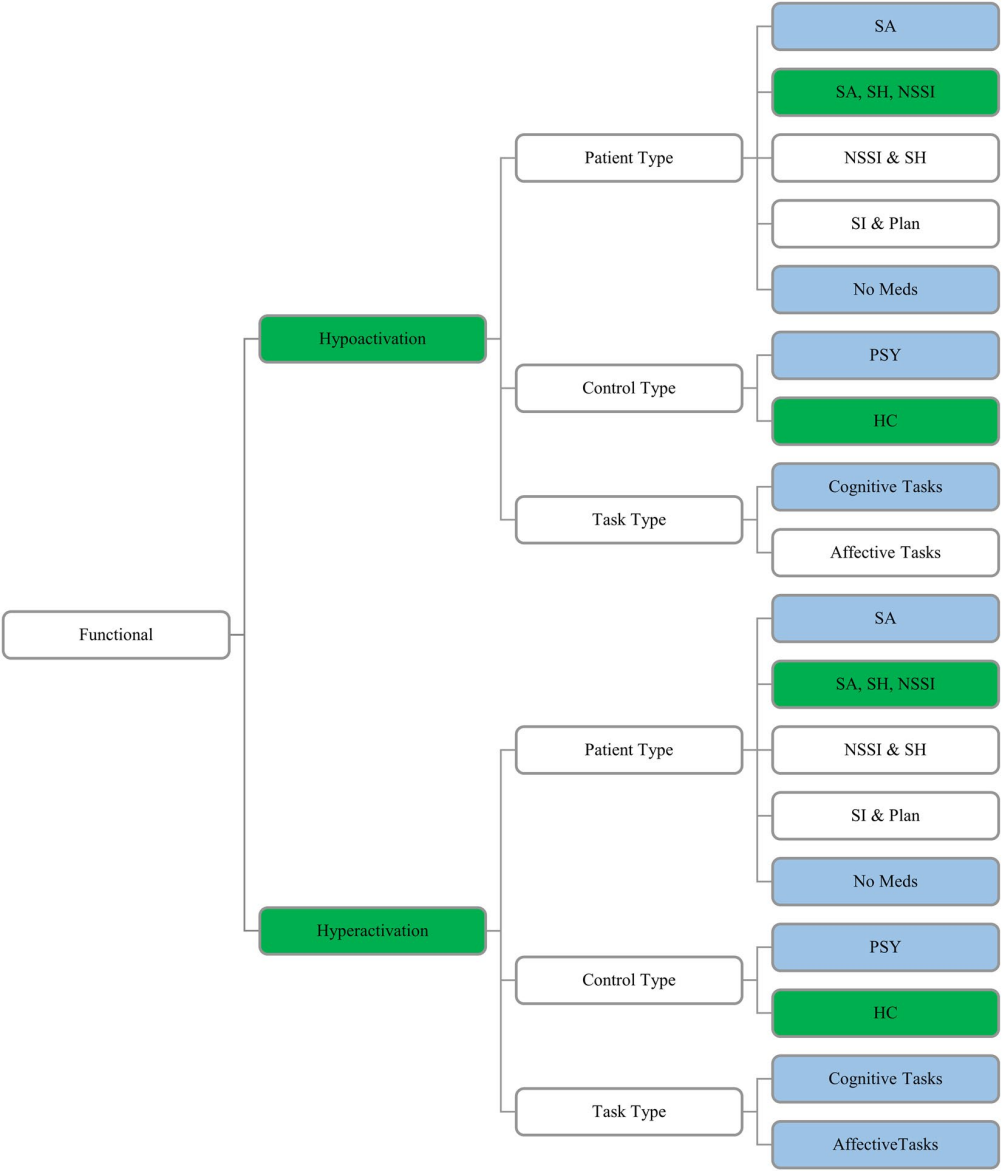
Breakdown of Functional Studies. Note. All possible contrasts from the meta-analysis are listed above. Those contrasts that contained 20 or more experiments are highlighted in green. Those contrasts that contained 10 or more experiments are highlighted in blue. HC: healthy controls; PSY: matched psychiatric populations; SITB: all self-injurious thoughts and behaviors; SA: patients with suicide attempts; SH: self-harm regardless of intent; NSSI: non-suicidal self-injury; SI: suicidal ideation.

**Figure 3:**
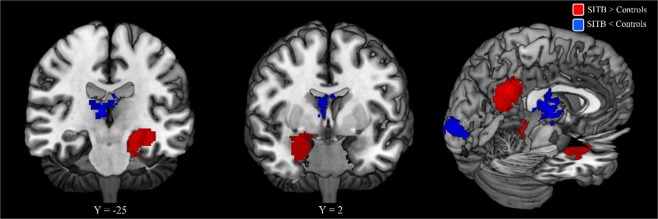
Results of Functional Imaging Studies from MKDA Method at 15 mm radii. Note. Significant results for SITB > Controls using MKDA (red) and SITB < Controls (blue) at 15 mm. Y coordinates are listed below the coronal sections. Results at 10 mm were clustered around the left calcarine and the left posterior cingulate cortex (PCC) with peak coordinates similar to the results shown above.

***Functional Imaging Studies***. No significant results were observed with any ALE analyses at either the standard 20 experiment criterion or the relaxed 10 experiment criterion (Fig. 2). However, MKDA with a 10 mm kernel requiring 20 experiments did reveal hyperactivation in SITBs at the left posterior cingulate cortex (PCC; Fig. 3 and Table 1), which was also observed when the kernel was increased to 15 mm. The right amygdala and the left hippocampus was also hyperactivated in SITBs at 15 mm (Fig. 3 and Table 1). In addition, hypoactivation in SITBs was observed in the right thalamus at 15 mm (Fig. 3 and Table 1).

**Table 1:** MKDA Coordinate Sites by Contrast for Functional Studies at 10 mm and 15 mm Radii with 20 Experiment Criterion.

Radius Size	Activation	Patient Type	Control Type	Number of Foci	Number of Cases	Number of Controls	Total Number of Subjects	Peak Coordinate (MNI)	Location	Number of Voxels	p*
10 m	Hypoactivation	SITBs	HCs	98	474	539	1013	−14, −98, 0	L calcarine	332	0.001
	Hyperactivation	SITBs	All Controls	263	789	813	1602	0, −56, 32	L PCC	352	<0.001
15 m	Hypoactivation	SITBs	All Controls	160	820	842	1662	2, −16, 10	R thalamus	642	0.001
		SITBs	HCs	98	474	539	1013	−14, −94, 4	L superior occipital gyrus	623	0.001
	Hyperactivation	SITBs	All Controls	263	789	813	1602	22, 0, −22 −24, −30, −18 0, −52, 34	R amygdala L hippocampus L PCC	686 488 1534	0.001 0.021 0.026
		SA,NSSI, SH	All Controls	123	452	479	931	22, 2, −24 −26, −26, −18 2, −56, 32	R amygdala L hippocampus L PCC	621 657 875	0.002 0.038 0.029
		SITBs	HCs	131	376	452	828	−32, 34, 34	Dorsolateral PFC	544	0.001

*Note*. SITBs: all self-injurious thoughts and behaviors; SA: suicide attempters; NSSI: individuals with non-suicidal self-injury; SH: individuals with self-harm behaviors regardless of intent; HC: healthy controls; R: right; L: left; PCC: posterior cingulate cortex; PFC: prefrontal cortex; *family-wise error corrected <0.05.

**Figure 4:**
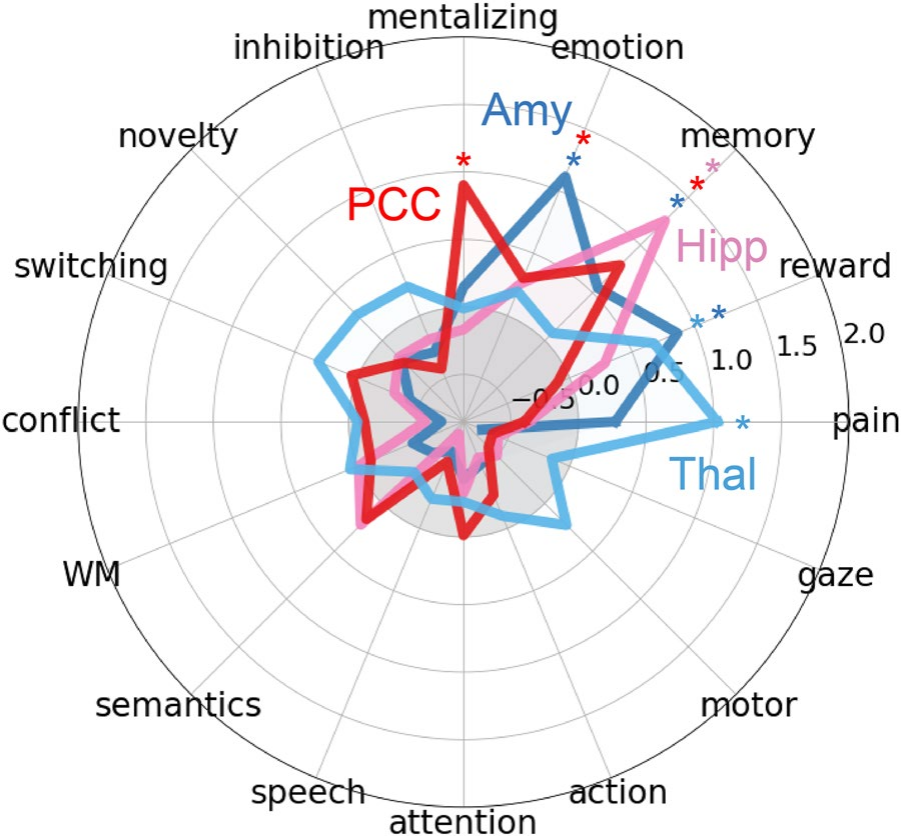
Functional Associations of Activation Sites. Note. Each cluster is illustrated to show which psychological concepts best predicted its activation based upon methods of de la Vega *et al*. (2017). Strength of association is measured in the log odds-ratio. Permutation-based significance after correction for multiple comparisons via false-discovery rate at q < 0.01 is indicated by an asterisk. PCC – posterior cingulate cortex; Amy – amygdala; Hipp – hippocampus; thal – thalamus.

***Functional Inference***. To infer the functional consequences of observed significant results, we performed functional specialization classification. Following the procedures of de la Vega and colleagues^31^, the psychological concepts that activate each significant cluster at radius 15 mm were inferred via machine learning classification on the NeuroSynth database^32^. The classification determines the extent to which psychological concepts predict activation of a given region. Areas of SITB-related hyperactivation (i.e., PCC, amygdala, and hippocampus) were significantly associated with internally-oriented processes of mentalizing, emotion, and memory, while SITB-related hypoactivation (i.e., thalamus) was significantly associated with pain (Fig. 4).

#### Meta-analyses for types of SITBs

To test the possibility that unique differences might be associated with specific types of SITBs, finer-grained meta-analyses were conducted provided sufficient power.

***Structural Imaging Studies***. Due to insufficient power, analyses could not be conducted for structural differences associated with specific types of SITBs with the stringent 20 experiment criterion (Fig. 1). Experiments reporting reduced volumes for suicide attempts and deliberate self-harm met the relaxed 10 experiment criterion, but neither ALE nor MKDA analyses yielded any significant findings.

***Functional Imaging Studies***. Experiments examining deliberate self-harm for both hypoactivation and hyperactivation findings met the stringent 20 experiment criterion (Fig. 2). ALE analyses did not produce a convergence in findings. MKDA with a 15 mm kernel, however, revealed a significant hyperactivation in the right amygdala, left hippocampus, and left PCC, a finding consistent with the pooled analysis (Table 1).

#### Moderator analyses

To investigate whether and how differences in samples and study designs might have affected findings, we attempted to conduct moderator analyses on type of control groups, psychiatric diagnoses, study paradigms, and medication status. However, moderator analyses could not be conducted for psychiatric diagnoses due to the heterogeneous inclusion criteria among studies. Other moderator analyses were conducted when they met either the more stringent minimum of 20 experiments or the more relaxed minimum of 10 experiments.

***Structural Imaging Studies***. No structural analyses met the stringent 20 experiment criterion (Fig. 1). There were sufficient experiments from MRI studies that reported less volume in the self-injurious participants compared to psychiatric controls to meet the relaxed 10 experiment criterion (Fig. 1). Consistent with the overall pooled analysis, neither ALE nor MKDA yielded any significant results.

***Functional Imaging Studies***. Regarding types of control groups, separate meta-analyses were conducted for both studies that used psychiatric controls and those that used healthy controls. Only experiments using healthy controls met the stringent 20 experiment criterion (Fig. 2). The rest of analyses were conducted with the minimum 10 experiment criterion. Consistent with the pooled analyses, ALE yielded no significant results. Inconsistent with the pooled analyses, MKDA with a 10 mm kernel for experiments with healthy controls only yielded significant hypoactivation in the left calcarine (Table 1), suggesting a moderator effect. For MKDA using a 15 mm kernel, a significant hypoactivation was observed in the left superior occipital gyrus and a significant hyperactivation was observed in the dorsolateral prefrontal cortex (PFC; Table 1). With the more relaxed 10 experiment criterion, MKDA with a 10 mm kernel revealed a significant hyperactivation was observed in the temporoparietal junction (TPJ) for experiments using psychiatric controls (Table 2).

**Table 2:** MKDA Coordinate Sites by Contrast for Studies at 10 mm and 15 mm Radii with 10 Experiment Criterion.

Radius Size	Activation	Patient Type	Control Type	Study Type	Number of Foci	Number of Cases	Number of Controls	Total Number of Subjects	Peak Coordinate (MNI)	Location	Number of Voxels	p*
10 m	Hyperactivation	SITBs	PSY	Functional Studies	97	284	249	533	−52, −66, 28	TPJ	438	0.001
15 m	Hyperactivation	SITBs	All Controls	Affective Tasks	128	301	292	593	4, −54, 34 −16, 26, 56	R PCC SFG	1127 683	0.003 0.047

*Note*. SITBs: all self-injurious thoughts and behaviors; PSY: matched psychiatric population; R: right; TPJ: temporoparietal junction; PCC: posterior cingulate cortex; SFG: superior frontal gyrus; *family-wise error corrected <0.05.

In terms of study paradigms, separate meta-analyses were conducted for experiments that used cognitive tasks or affective tasks with a relaxed 10 experiment criterion. Analyses yielded no significant findings for cognitive tasks. For affective tasks, ALE did not yield any significant findings. MKDA with a 15 mm kernel showed significant hyperactivation in the right PCC and superior frontal gyrus (SFG), indicating a moderator effect (Table 2). This finding was not replicated for MKDA with a 10 mm kernel.

With respect to medication status, no analyses met the stringent 20 experiment minimum (Fig. 2). Using the 10 experiment criterion, neither ALE nor MKDA yielded any significant results when only non-medicated individuals were included.

## Discussion

The present study yielded four major findings: (1) existing neuroimaging research has not found consistent structural brain differences between populations with and without SITBs; (2) the ALE method produced no significant findings regarding functional differences, while in most inclusive analysis, the MKDA method produced a convergence of findings at four locations (i.e., left PCC, right thalamus, right amygdala, and left hippocampus); (3) finer-grained meta-analyses for specific types of SITBs showed that deliberate self-harm might be associated with functional differences in the right amygdala, left hippocampus, and left PCC; and (4) moderator analyses showed checkered consistency. These findings together suggest that the extant literature provides some, but far from unanimous, support for neural correlates of SITBs. The major findings and the strengths and limitations of the present study are discussed in more detail below.

Despite previous research suggesting structural differences associated with SITBs^18,20^, the present meta-analysis did not yield any significant findings with either ALE or MKDA. To our surprise, the meta-analysis did not replicate previous reviews suggesting distinct structural changes associated with SITBs^21,22^. A primary difference between this study and previous reviews is that we subscribed to power guidelines in the field^28^ that required a more stringent minimum of 20 experiments for each analysis. Even though we conducted additional analyses with a more relaxed 10 experiment criterion, more studies are needed to reveal the consistent differences between individuals with and without SITBs. Due to the insufficient number of studies in the literature, it is unclear whether certain structural differences are associated with risk for SITBs in general or for specific types of SITBs. Additionally, it is possible that certain factors moderate structural findings but the limited number of studies prevented us from detecting such effects. For instance, it is unclear whether structural differences associated with SITBs might be primarily manifested in structural connectivity rather than regional volumes. Similarly, it is unclear whether thickness or surface areas might be more relevant to SITBs than volumes. Some researchers further suggest that structural differences might be particularly pronounced among suicide attempters who employed violent methods^26^. Lastly, in spite of multiple potential explanations for the current null findings, we cannot rule out the possibility that individuals with SITBs might not exhibit consistent structural differences.

Our second major finding is that, when all types of control groups were considered, functional differences in the left PCC, right thalamus, right amygdala, and left hippocampus were associated with SITBs based on the MKDA method, but not the ALE method. Many of these regions have not been a focus of the literature on SITBs. If the locations indeed reflect underlying brain differences associated with SITBs, future research might benefit from examining these areas in more detail. The most robust finding across analyses was hyper-activation of the PCC in individuals with SITBs. The PCC is one of the major nodes of default network, which is engaged during mind-wandering, and projects heavily to the memory system^33–35^. Consistent with its role in the default network, functional profiling of the PCC revealed strong associations with mentalizing, emotion, and memory. Similarly, the hippocampus and amygdala were also shown to be hyper-active in individuals with SITBs and also have well-established roles in memory and emotion^36–38^. Collectively, these data suggest that across functional tasks, individuals with SITBs showing a greater propensity for internally-directed processing. On the other hand, MDKA revealed some evidence of hypo-activation of thalamic areas involved in pain in individuals with SITBs. Together, a propensity for internally-oriented, emotional processing coupled with under-active pain processing could form the basis of SITBs. The lack of convergence between the two conceptually similar analyses (i.e., ALE and MKDA), however, leads to questions about the robustness of the findings. More studies are needed to shed light on this topic.

Our third major finding is that the MKDA method, but not the ALE method, found associations between deliberate self-harm and the left PCC, right amygdala, and left hippocampus. We originally intended to conduct separate meta-analyses for each type of SITBs. To our surprise, the majority of the literature focused on studying suicide attempts, with much less focus on suicide ideation, plan, and NSSI. Therefore, we were only able to perform analyses for suicide attempts and deliberate self-harm. Consistent with the pooled analysis of all SITBs, the MKDA with a 15 mm kernel revealed significant hyperactivation in the left PCC, right amygdala, and left hippocampus for deliberate self-harm. It is possible that brain differences might be associated with general risk for SITBs instead of specific types of SITBs. Therefore, differences associated with self-harm regardless of intent might be more consistent with the overall finding than only self-harm with the intent to die (i.e., suicide attempt). It is also possible that the inclusion of self-harm without intent to die simply boosted the power to detect a significant difference. Given the paucity of research on certain types of SITBs, however, it is unclear whether unique brain changes exist for specific types of SITBs or whether they are associated with a general risk for SITBs. More studies examining self-injurious phenomena other than suicide attempts are needed to provide further insight on this issue.

Lastly, moderator analyses demonstrated checkered consistency for the significant functional differences yielded by the pooled meta-analyses. Even though it was within our initial intention to systematically conduct moderator analyses, the unexpectedly limited number of experiments within each moderator category prevented us from fully performing these analyses. However, within the constraints of the literature, we conducted all moderator analyses that met the more relaxed 10 experiment criterion. Significant moderator effects of type of control groups and study paradigms were detected from the MKDA method. However, these moderator effects were not robust as the results were generated from less stringent analyses and inconsistent across MKDA and ALE. More studies are needed detect consistent moderator effects.

The present findings should be considered within the context of the study’s limitations. It is important to note that a meta-analysis summarizes and reflects the current state of the literature and is therefore largely constrained by the limitations of the literature. First, the statistical power of the present meta-analysis was confined both in terms of the number of experiments and participants. It was surprising that few structural findings met the new power guidelines in the field^28^, and a limited number of functional findings did. Regarding sample size, It is generally well appreciated that studies with a small sample size might lack the statistical power to detect true effects; however, small sample size also reduces the likelihood that a detected result reflects a true effect^39^. The median sample size of the studies included in the meta-analysis is 48, which can lead to poor replicability even in one-sample tests^40^, let alone two-sample tests. On the other hand, increasing sample size will only improve power if there is an underlying group-level effect to find. Recent data indicate that there are multiple neurophysiological subtypes of depression^41^, and it is possible that SITBs are just as, or even more variable. Furthermore, group-level inferences may not apply to individuals^42^. Such data suggest that more data are needed at the individual-level. Hence, insufficient power at either the group- and/or individual-levels may contribute to the inconsistent findings in the present meta-analysis.

Second, the heterogeneity among studies in the literature might have obscured the meta-analytic findings. For example, the thresholds that studies set to control for multiple comparisons vary widely. Insufficiently corrected analyses produce false positives, adding noise to meta-analyses^43^. Similarly, high heterogeneity exists regarding preprocessing parameters and the contrasts analyzed, which can lead to vastly different results on the same underlying data^44^. Moreover, 40.25% of the contrasts used healthy controls instead of psychiatric controls to test for differences associated with SITBs. Considering that individuals with SITBs are likely to meet diagnostic criteria for psychiatric conditions, psychiatric controls would provide a more stringent comparison and reduce the likelihood of detecting differences associated with general psychopathology instead of SITBs. Further, even though the present study was unable to directly examine the effects of specific psychological tasks (e.g., Iowa Gambling Task, Stroop task) employed in the studies due to insufficient statistical power, it is possible that these differences contributed to the checkered consistency of the findings. Of note, heterogeneity and flexibility in study paradigms and analytical decisions might have also obscured previous reviews and meta-analyses, contributing to the mixed conclusions in the literature.

In addition to limitations of the literature, it is important to keep in mind limitations of coordinate-based meta-analysis (CBMA). CBMA has been used to identify convergent activations in numerous domains^45–48^, but it remains an imperfect method. CBMA creates simulated statistical maps based upon peak activations reported in studies. However, the size and shape of the simulated activation clusters are unrealistic, potentially leading to both false positive and negative results. It would also be prudent to weight activation clusters by their effect size^49^. However, effect size information was irregularly reported in the present sample. In an ideal scenario, meta-analysis of neuroimaging data would be performed on unthresholded statistical maps, which would at once provide size, shape, and effect size estimates. Although resources such as NeuroVault are becoming increasingly popular^50^, they are not yet used widely enough to perform meta-analyses in this domain. More consistent data sharing in the future would help to determine whether the present findings were due to limitations of CBMA.

Despite the limitations, this study also demonstrates several strengths. First, even though this meta-analysis was still underpowered for some sub-analyses and moderator analyses, it demonstrates one of the largest efforts to increase power to detect true underlying effects by including neuroimaging studies on any type of SITBs. Second, the meta-analysis employed two gold-standard coordinate-based meta-analytic methods (i.e., ALE and MKDA). This signaled progress over meta-analyses that largely relied on ALE alone and allowed for evaluation of the robustness of findings. Third, we subscribed to the new power guideline in the field^28^, but also conducted analyses using the previous criterion for completeness^51^. The power standard (i.e., a minimum of 20 experiments per analysis) is considered to be more stringent than previous standards. By following these guidelines, the present study is less likely to yield spurious findings.

To summarize, the present meta-analysis aimed to evaluate the *current* empirical evidence for neural correlates of SITBs and whether it justifies any definitive conclusions about brain differences among individuals with SITBs. This study conducted pooled analyses across all SITBs, separate analyses for specific types of SITBs, and moderator analyses of differences among studies. The current state of the literature failed to provide support for structural differences, and provided some, yet far from unequivocal, support for functional differences. The identified differences in the left PCC, right thalamus, right amygdala, and left hippocampus have not been the focus of previous studies, but may offer promising future avenues of exploration. Due to the constraints of the existing literature, it is unclear whether brain differences increase general risk for SITBs or unique differences are associated with specific types of SITBs. Insufficient power, heterogeneity in study paradigm, flexibility in analytical decisions, and limitations of CBMA might have hindered the current study from identifying consistent and robust patterns associated with SITBs. Given the extant literature, more studies are needed to reach definitive conclusions on differences in brain structure and function among people with a history of SITBs. Future studies should consider gathering more group and/or individual-level data, selecting stringent control groups, providing replications of previous research, and adopting standard thresholds and preprocessing parameters.

## Methods

### Literature search and inclusion criteria

We identified relevant articles using a range of search terms through January 1, 2019 using PubMed, PsycInfo, and Google Scholar. We intentionally chose a large number of search terms to increase likelihood of identifying relevant articles that may have been missed otherwise. Search terms included variants of “suicide”, “self-injury”, “self-harm”, “self-directed violence”, “self-mutilation”, “deliberate self-harm (DSH)”, and “nonsuicidal self-injury (NSSI)” crossed with variants of “computerized tomography (CT)”, “magnetic resonance imaging (MRI)”, positron emission tomography (PET)”, “single-photon emission computed tomography (SPECT)”, “diffusion tensor imaging (DTI)”, “magnetic resonance spectroscopy (MRS)”, “neuroimaging”, “gray matter”, “white matter”, “serotonin”, “dopamine”, “gamma-aminoburtyric acid”, “noradrenaline”, “norepinephrine”, and “brain”.

Inclusion required that studies (1) include at least one group of which all individuals exhibit SITBs; (2) include at least one control group; (3) conduct whole-brain analyses; and (4) provide standardized coordinates. The first inclusion criterion is to ensure that the findings are uniquely associated with SITBs instead of general psychopathology, with the second criterion ensuring that each study provided a benchmark for comparison. The third criterion is to prevent Region of Interest (ROI) analyses from biasing the meta-analytic results. Although ROI analyses provide valuable information about the neural correlates of SITBs, they violate the assumption that each voxel has an equal chance of being activated, thus biasing the meta-analytic results toward convergence on the ROIs^52^. This inclusion criterion is consistent with other meta-analyses of brain imaging studies^30,53^.

**Figure 5:**
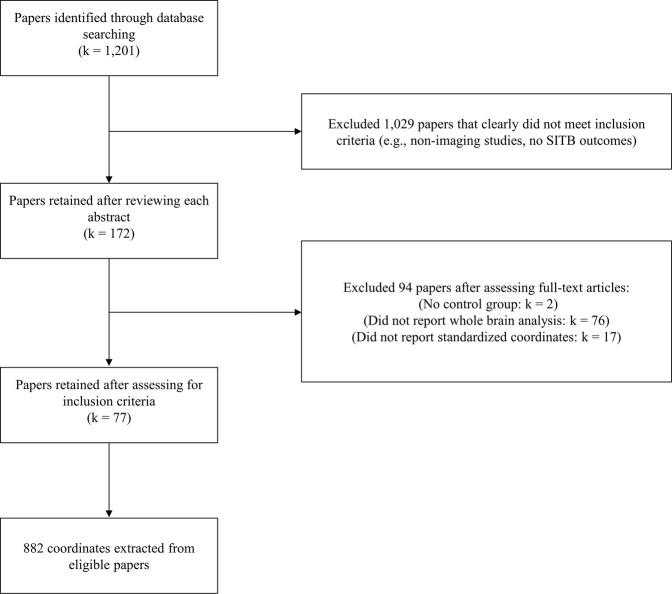
PRISMA Flowchart. Note. k = number of papers.

A total of 1,201 unique papers were identified through database searching. Seventy-seven papers were retained in the present study, yielding a total sample size of 4,903 participants. To reduce nonindependence of multiple papers published on the same samples, only findings from unique contrasts were extracted. For instance, if analyses were first conducted on the entirety of the sample and subsequently repeated on subsamples, only findings from the broader analyses were included as they represented the most inclusive data. A total of 882 unique coordinates were extracted (see Fig. 5 for PRISMA flowchart, Supplement 1 for a list of included studies, and Supplement Table S1 for description of the studies and contrasts).

### Data extraction

We extracted the following information from each study: (1) sample size; (2) imaging techniques; (3) type of SITBs; (4) type of control groups (i.e., self-injurious, psychiatric, healthy controls); (5) psychiatric diagnoses; (6) Montreal Neurological Institute (MNI) or Talaraich coordinates; (7) study paradigm (i.e., resting-state versus task-based, with tasks further divided into cognitive versus affective tasks); (8) sample age, and (9) sample medication status.

#### Sample size

We extracted the sample size associated with each contrast.

#### Imaging techniques

Consistent with previous reviews and meta-analysis, we included studies with a range of imaging techniques^5,8,21–23,27^. The types of imaging techniques were determined from each study: Magnetic Resonance Imaging (MRI), functional Magnetic Resonance Imaging (fMRI), Diffusion Tensor Imaging (DTI), Positron Emission Tomography (PET), and Single-Photon Emission Computed Topography (SPECT).

#### Type of SITBs

We adhered to the terminology proposed by Nock^2^ and categorized SITBs examined by each study into: suicide ideation, suicide plan, suicide attempt, suicide death, and nonsuicidal self-injury (NSSI). When a study examined deliberate self-injuries of which the intent to die was unclear, we labeled the type of SITBs as self-harm. When a study examined suicide attempt and other suicidal behaviors (e.g., interrupted attempt, aborted attempt) together, the study was considered to have examined all suicidal behaviors. We intentionally included all types of SITBs to conduct a pooled meta-analysis as well as finer-grained analyses to test whether certain brain differences are associated with general risk for SITBs or only specific types of SITBs.

#### Type of control groups

A control group was considered a self-injurious control if participants were selected based on prior or current SITBs (e.g., suicide ideation, NSSI). A control group was coded as a psychiatric control if participants were drawn because they met certain clinical conditions (e.g., a psychiatric diagnosis, a score above the clinical threshold on a measure). When neither eligibility criteria were set by the study, the control group was considered as a healthy control. This code was intended to test whether the stringency of control group might have contributed to the diverse findings in the literature.

#### Psychiatric diagnoses

Given that some evidence suggests that the brain differences associated with SITBs might vary depending on the psychiatric diagnoses^15,54^, we coded for the primary psychiatric diagnoses of the samples.

#### Montreal neurological institute (MNI) or talairach coordinates

Whenever provided, MNI or Talairach coordinates were directly extracted for each contrasts from the studies. If a study did not specify whether they provided MNI or Talairach coordinates, the type of coordinates were inferred based on the statistical software used by the authors.

#### Study paradigm

Following convention of prior reviews and meta-analyses^5,8,21–23,27^, we included studies using a wide range of study paradigms. To estimate and control for differences between studies, we first categorized each contrast based on whether they were obtained via resting-state or task-based paradigms. We then categorized task-based paradigms into cognitive tasks, affective tasks, tasks involving pain, and other tasks (e.g., motor tasks). Based on both convention in the field and descriptions provided within each study, all tasks reported by studies could be categorized into one of the four categories. For instance, Tower of London Test, Go/No-Go Task, N-Back Task, and Continuous Performance Task were categorized as cognitive tasks. Examples of affective tasks include viewing pictures with negative valence, matching emotional faces, and tasks inducing social rejection. Given an insufficient number of coordinates reported from tasks other than cognitive and affective tasks, they could not be meta-analyzed as a separate category. Therefore, separate analyses were only conducted for cognitive and affective tasks. Even though it was our original intention to code for specific tasks (e.g., Stroop task, Iowa Gambling Tasks) and to test whether they moderate the findings, we were unable conduct such analyses due to heterogeneity in the literature. Despite the fact that non-neuroimaging meta-analyses have analyzed these tasks when there were at least three studies using the same task^55^, guidelines suggest a minimum of 20 experiments in each category for coordinate-based meta-analyses^28^. As such, we were unable to produce finer-grained categorizations.

#### Sample age

The mean sample age was extracted from each contrast. We also categorized sample age into adult, adolescent, elderly, and mixed samples. A sample was coded as adult if all the participants were at least 18 years old but less than 65 years old, and elderly if all the participants were at least 65 years old. A sample was coded as adolescent if all the participants were under the age of 18. When a sample included both adult and adolescent participants, it was coded as mixed adult and adolescent. Similarly, when a sample included both adult and elderly participants, it was coded as mixed adult and elderly.

#### Medication status

To assess for potential moderator effects, samples were coded into the following categories based on participants’ psychiatric medication status: none medicated, at least some medicated, or all medicated.

### Statistical analysis

The goal of this meta-analysis was to identify brain areas that were consistently related to SITBs. We addressed this goal using two methods of coordinate-based meta-analysis (CBMA): Activation Likelihood Estimation (ALE^56–58^) and Multi-level Kernel Density Analysis (MKDA^59^). Ideally, meta-analysis of neuroimaging data would be performed on statistical maps. Unfortunately, such maps are rarely available despite current efforts to create map repositories^50,60^. In lieu of such maps, CBMA infers statistical maps based upon locations of statistical local maxima (i.e. peaks). Then, spatial-consistency among the inferred statistical maps is assessed. As detailed below, the two methods employed here differ in how the inferred statistical maps are calculated. The use of two different meta-analytic procedures was to ensure that the results did not depend on methodological specifics. To control for multiple comparisons and to reduce Type I error, statistical significance in both meta-analytic methods was determined using cluster-level family-wise error (FWE) correction, as has been recommended for CBMA^28^. Cluster extents were determined at a height threshold of *p* < 0.001 using Monte-Carlo permutation methods^59,61^.

To address heterogeneity in study methods, meta-analyses followed a tree approach to assess structural (Fig. 1) and functional (Fig. 2) differences between populations with and without SITBs. First, to assess for general brain differences that might predispose all individuals to all types of SITBs, we conducted pooled analyses that included all SITBs and all types of sample populations. Second, to test for specific brain changes associated with specific types of SITBs, we conducted separate analyses for each type of SITBs when power allowed. Lastly, to test for potential moderator effects, we conducted more granular analyses based on control group type, psychiatric diagnoses, task type, and medication status, provided sufficient power. We subscribed to the power guidelines proposed by Eickhoff and colleagues^28^, which required a minimum of 20 experiments in each category for ALE. Therefore, in our main report, we focused on those analyses that had 20 or more experiments. For completeness, we reported analyses with at least 10 experiments in Table 2, which had been an earlier criterion^51^.

#### Activation likelihood estimation (ALE): overview

ALE was performed using GingerALE software version 2.3^56–58^. For each experiment, ALE computes “modeled activation” maps that indicate the probability that a given voxel was “activated.” (Although “activated” suggests a functional change, the same logic can be applied to structural data. We use the term “activated” for convenience and consistency with the methodological reports). ALE assumes that each peak represents a broader activation cluster and that the exact location of each peak/cluster is uncertain. Therefore, each peak is convolved with a Gaussian kernel to form a Gaussian probability density. The kernel has a fixed area-under-the-curve, but the full-width/half-maximum (FWHM) varies according to the sample size of the experiment, with the FWHM values empirically determined^58^. This results in narrower, higher amplitude peak densities for large sample sizes reflecting greater certainty, and broader, lower amplitude peak densities for small sample sizes reflecting less certainty. To control for the fact that studies vary in whether or not sub-peaks within a cluster are reported, we used the non-additive approach that assigns the maximal density amplitude to a voxel that is activated across multiple clusters^57^. ALE values are computed for each voxel via the voxel-wise union of the modeled activation maps. Observed ALE values are then compared to a randomly permuted null distribution to determine significance^61^. Cluster extents were determined at a height threshold of *p* < 0.001 using previously recommended Monte-Carlo permutation methods of 1000 permutations^61^.

#### Multi-level kernel density analysis (MKDA): overview

MKDA^59^ differs from ALE in terms of the kernel that is convolved with each activation peak. MKDA uses a spherical kernel whose radius is determined by the analyst, whereas ALE uses a Gaussian kernel whose FWHM is empirically determined. At first blush, an empirically determined kernel extent may seem superior to an arbitrarily assigned kernel extent. However, the empirically determined FWHM is based upon data from 21 healthy participants performing a single task with BOLD imaging^58^. Whether the extents observed there generalize to different populations, imaging modalities, and tasks is unclear. Therefore, the ability to freely choose a kernel extent in MKDA offers assurance that significant/non-significant results are not due to this limitation.

We conducted analyses with kernel radii at 10 mm and 15 mm, which has been previously recommended^48,59^. For each study, each peak was convolved with the kernel to create a comparison indicator map. The map has values of either 1 (‘a study activated near this voxel’) or 0 (‘a study did not activate near this voxel’). Similar to the non-additive approach to ALE, the nesting of peaks within studies allows that no one study can disproportionally contribute to the significant findings. Each map is weighted by the product of the square root of the study sample size. The weighted average of these maps is then compared to a randomly permuted null distribution to determine significance. Cluster extents were determined at a height threshold of *p* < 0.001 using the previously recommended Monte-Carlo permutation methods of 5000 permutations^59^.

#### Sample size determination

Both ALE and MKDA weight studies by sample size. Both methods were developed with one-sample tests in mind and thus the weighting procedures assume a one-sample *n*. Here, we are explicitly focused on two-sample tests. To provide an equivalent one-sample *n* we used the equation (*n*_1_ × *n*_2_)/(*n*_1_ + *n*_2_) following prior guidelines^49^.

#### Spatial distributions

Both ALE and MKDA use Monte-Carlo procedures to determine the null distribution. By default, both ALE and MKDA restrict random permutation to gray matter. However, this procedure is not appropriate for analyses that are expected to produce results in white matter (e.g. DTI). Therefore, for MKDA analyses of DTI data, we used a white matter, rather than gray matter mask.

## Supplementary information

Supplemental Information.

Dataset 1.

## Data Availability

The datasets analyzed during the current study are available from the corresponding author on reasonable request.
