# Supplementary material for: Brain Differences Associated with Self-Injurious Thoughts and Behaviors: A Meta-Analysis of Neuroimaging Studies
Source: Sci Rep. 2020 Feb 12;10:2404. doi: 10.1038/s41598-020-59490-6 (PMC7016138; doi:10.1038/s41598-020-59490-6)
Supplement: Supplementary file 1 — Supplemental Information. [file 41598_2020_59490_MOESM1_ESM.docx]

**Brain Differences Associated with Self-Injurious Thoughts and Behaviors:**

**A Meta-Analysis of Neuroimaging Studies**

Xieyining Huang, MS,^1*^ Kelly Rootes-Murdy, MS, ^1,2^ Diana M. Bastidas, BS, ^1^

Derek E. Nee, PhD, ^1^ Joseph C. Franklin, PhD^1^

^1^Department of Psychology, Florida State University, Tallahassee, Florida, USA

^2^Department of Psychology, Georgia State University, Atlanta, Georgia, USA

* Address correspondence to: Xieyining Huang, Department of Psychology, Florida State University, 1107 W Call St, Tallahassee, FL, 32306; Phone: (850) 644-2040; Fax: (850) 644-7739; [huang@psy.fsu.edu](mailto:huang@psy.fsu.edu).

**Keywords:** Meta-analysis; neuroimaging; suicide; self-injury; brain.

**Supplemental Information**

**Supplement 1.** List of Included Studies.

**Table S1.** Description of Included Studies.

**Supplement 1. List of Included Studies.**

1. Aguilar, E. J. *et al.* Left orbitofrontal and superior temporal gyrus structural changes associated to suicidal behavior in patients with schizophrenia. *Prog. Neuro-Psychopharmacology Biol. Psychiatry* **32**, 1673–1676 (2008).

2. Ai, H. *et al.* Differential relations of suicidality in depression to brain activation during emotional and executive processing. *J. Psychiatr. Res.* **105**, 78–85 (2018).

3. Alarcón, G., Sauder, M., Teoh, J. Y., Forbes, E. E. & Quevedo, K. Amygdala functional connectivity during self-face processing in depressed adolescents with recent suicide attempt. *J. Am. Acad. Child Adolesc. Psychiatry* **58**, 221–231 (2019).

4. Ambrosi, E. *et al.* Resting-state functional connectivity of the habenula in mood disorder patients with and without suicide-related behaviors. *J. Neuropsychiatry Clin. Neurosci.* **31**, 49–56 (2018).

5. Amen, D. G., Prunella, J. R., Fallon, J. H., Amen, B. & Hanks, C. A comparative analysis of completed suicide using high resolution brain SPECT Imaging. *J. Neuropsychiatry Clin. Neurosci.* **21**, 430–439 (2009).

6. Audenaert, K. *et al.* SPECT neuropsychological activation procedure with the Verbal Fluency Test in attempted suicide patients. *Nucl. Med. Commun.* **23**, 907–916 (2002).

7. Beauchaine, T. P., Sauder, C. L., Derbidge, C. M. & Uyeji, L. L. Self-injuring adolescent girls exhibit insular cortex volumetric abnormalities that are similar to those seen in adults with borderline personality disorder. *Dev. Psychopathol.* **31**, 1203–1212 (2018).

8. Benedetti, F. *et al.* Opposite effects of suicidality and lithium on gray matter volumes in bipolar depression. *J. Affect. Disord.* **135**, 139–147 (2011).

9. Benedetti, F. *et al.* The serotonin transporter genotype modulates the relationship between early stress and adult suicidality in bipolar disorder. *Bipolar Disord.* **16**, 857–866 (2014).

10. Besteher, B. *et al.* Pronounced prefronto-temporal cortical thinning in schizophrenia: Neuroanatomical correlate of suicidal behavior? *Schizophr. Res.* **176**, 151–157 (2016).

11. Bijttebier, S. *et al.* The vulnerability to suicidal behavior is associated with reduced connectivity strength. *Front. Hum. Neurosci.* **9**, 1–11 (2015).

12. Brown, R. C. *et al.* Differential neural processing of social exclusion and inclusion in adolescents with non-suicidal self-injury and young adults with borderline personality disorder. *Front. Psychiatry* **8**, 267 (2017).

13. Cao, J. *et al.* Abnormal regional homogeneity in young adult suicide attempters with no diagnosable psychiatric disorder: A resting state functional magnetic imaging study. *Psychiatry Res. - Neuroimaging* **231**, 95–102 (2015).

14. Cao, J. *et al.* Resting-state functional MRI of abnormal baseline brain activity in young depressed patients with and without suicidal behavior. *J. Affect. Disord.* **205**, 252–263 (2016).

15. Chase, H. W. *et al.* Alterations of functional connectivity and intrinsic activity within the cingulate cortex of suicidal ideators. *J. Affect. Disord.* **212**, 78–85 (2017).

16. Chen, Z. *et al.* Magnetization transfer imaging of suicidal patients with major depressive disorder. *Sci. Rep.* **5**, 1–6 (2015).

17. Davis, T. S. *et al.* Emotional reactivity and emotion regulation among adults with a history of self-harm: Laboratory self-report and functional MRI evidence. *J. Abnorm. Psychol.* **123**, 499–509 (2014).

18. Ding, Y. *et al.* Altered brain processing of decision-making in healthy first-degree biological relatives of suicide completers. *Mol. Psychiatry* **22**, 1149–1154 (2016).

19. Fan, T., Wu, X., Yao, L. & Dong, J. Abnormal baseline brain activity in suicidal and non-suicidal patients with major depressive disorder. *Neurosci. Lett.* **534**, 35–40 (2013).

20. Fan, S. *et al.* Gray and white matter differences in adolescents and young adults with prior suicide attempts across bipolar and major depressive disorders. *J. Affect. Disord.* **245**, 1089–1097 (2019).

21. Fradkin, Y., Khadka, S., Bessette, K. L. & Stevens, M. C. The relationship of impulsivity and cortical thickness in depressed and non-depressed adolescents. *Brain Imaging Behav.* 1–11 (2016). doi:10.1007/s11682-016-9612-8

22. Garrion, V. G., Garrett, A., Menon, V., Weems, C. F. & Reiss, A. L. Posttraumatic stress symptoms and brain function during a response-inhibition task: An fMRI study in youth. *Depress. Anxiety* **25**, 514–526 (2008).

23. Groschwitz, R. C., Plener, P. L., Groen, G., Bonenberger, M. & Abler, B. Differential neural processing of social exclusion in adolescents with non-suicidal self-injury: An fMRI study. *Psychiatry Res. - Neuroimaging* **255**, 43–49 (2016).

24. Harenski, C. L., Harenski, K. A., Calhoun, V. D. & Kiehl, K. A. Source-based morphometry reveals gray matter differences related to suicidal behavior in criminal offenders. *Brain Imaging Behav.* 1–9 (2018).

25. Hwang, J.-P. *et al.* Cortical and subcortical abnormalities in late-onset depression with history of suicide attempts investigated with MRI and voxel-based morphometry. *J. Geriatr. Psychiatry Neurol.* **23**, 171–184 (2010).

26. Jia, Z. *et al.* High-field magnetic resonance imaging of suicidality in patients with major depressive disorder. *Am. J. Psychiatry* **167**, 1381–1390 (2010).

27. Johnston, J. A. Y. *et al.* Multimodal neuroimaging of frontolimbic structure and function associated with suicide attempts in adolescents and young adults with bipolar disorder. *Am. J. Psychiatry* **174**, 667–675 (2017).

28. Jollant, F. *et al.* Orbitofrontal cortex response to angry faces in men with histories of suicide attempts. *Am. J. Psychiatry* **165**, 740–748 (2008).

29. Jollant, F. *et al.* Decreased activation of lateral orbitofrontal cortex during risky choices under uncertainty is associated with disadvantageous decision-making and suicidal behavior. *Neuroimage* **51**, 1275–1281 (2010).

30. Just, M. A. *et al.* Machine learning of neural representations of suicide and emotion concepts identifies suicidal youth. *Nat. Hum. Behav.* 1–9 (2017). doi:10.1038/s41562-017-0234-y

31. Kang, S.-G. *et al.* Resting-state functional connectivity of the amygdala in suicide attempters with major depressive disorder. *Prog. Neuro-Psychopharmacology Biol. Psychiatry* **77**, 222–227 (2017).

32. Kim, Y. J. *et al.* A pilot study of differential brain activation to suicidal means and DNA methylation of CACNA1C gene in suicidal attempt patients. *Psychiatry Res.* **255**, 42–48 (2017).

33. Kraus, A. *et al.* Script-driven imagery of self-injurious behavior in patients with borderline personality disorder: A pilot FMRI study. *Acta Psychiatr. Scand.* **121**, 41–51 (2010).

34. Lan, M. J. *et al.* Resting-state amplitude of low-frequency fluctuation is associated with suicidal ideation. *Depress. Anxiety* **36**, 433–441 (2019).

35. Lee, K. H. *et al.* Self-harm in schizophrenia is associated with dorsolateral prefrontal and posterior cingulate activity. *Prog. Neuro-Psychopharmacology Biol. Psychiatry* **61**, 18–23 (2015).

36. Lee, S. J. *et al.* White matter alterations associated with suicide in patients with schizophrenia or schizophreniform disorder. *Psychiatry Res. - Neuroimaging* **248**, 23–29 (2016).

37. Lee, Y. J. *et al.* Decreased regional gray matter volume in suicide attempters compared to suicide non-attempters with major depressive disorders. *Compr. Psychiatry* **67**, 59–65 (2016).

38. Leyton, M. *et al.* α-[11C]methyl-L-tryptophan trapping in the orbital and ventral medial prefrontal cortex of suicide attempters. *Eur. Neuropsychopharmacol.* **16**, 220–223 (2006).

39. Li, J., Duan, X., Cui, Q., Chen, H. & Liao, W. More than just statics: Temporal dynamics of intrinsic brain activity predicts the suicidal ideation in depressed patients. *Psychol. Med.* **49**, 852–860 (2019).

40. Lippard, E. T. C. *et al.* Preliminary examination of gray and white matter structure and longitudinal structural changes in frontal systems associated with future suicide attempts in adolescents and young adults with mood disorders. *J. Affect. Disord.* **245**, 1139–1148 (2019).

41. Long, Y. *et al.* Associations among suicidal ideation, white matter integrity and cognitive deficit in first-episode schizophrenia. *Front. Psychiatry* **9**, 391 (2018).

42. Mahon, K., Burdick, K. E., Wu, J., Ardekani, B. A. & Szeszko, P. R. Relationship between suicidality and impulsivity in bipolar I disorder: A diffusion tensor imaging study. *Bipolar Disord.* **14**, 80–89 (2012).

43. Marchand, W. R. *et al.* Striatal and cortical midline circuits in major depression: Implications for suicide and symptom expression. *Prog. Neuro-Psychopharmacology Biol. Psychiatry* **36**, 290–299 (2012).

44. Matthews, S., Spadoni, A., Knox, K., Strigo, I. & Simmons, A. Combat-exposed war veterans at risk for suicide show hyperactivation of prefrontal cortex and anterior cingulate during error processing. *Psychosom. Med.* **74**, 471–475 (2012).

45. Miller, A. B. *et al.* Neural correlates of emotion regulation and adolescent suicidal ideation. *Biol. Psychiatry Cogn. Neurosci. Neuroimaging* **3**, 125–132 (2018).

46. Minzenberg, M. J. *et al.* Control-related frontal-striatal function is associated with past suicidal ideation and behavior in patients with recent-onset psychotic major mood disorders. *J. Affect. Disord.* **188**, 202–209 (2015).

47. Minzenberg, M. J. *et al.* Conflict-related anterior cingulate functional connectivity is associated with past suicidal ideation and behavior in recent-onset schizophrenia. *J. Psychiatr. Res.* **65**, 95–101 (2015).

48. Minzenberg, M. J., Lesh, T. A., Niendam, T. A., Cheng, Y. & Carter, C. S. Conflict-related anterior cingulate functional connectivity is associated with past suicidal ideation and behavior in recent-onset psychotic major mood disorders. *J. Neuropsychiatry Clin. Neurosci.* **28**, 299–305 (2016).

49. Niedtfeld, I. *et al.* Affect regulation and pain in borderline personality disorder: A possible link to the understanding of self-injury. *Biol. Psychiatry* **68**, 383–391 (2010).

50. Niedtfeld, I. *et al.* Functional connectivity of pain-mediated affect regulation in borderline personality Disorder. *PLoS One* **7**, 1–10 (2012).

51. Olié, E. *et al.* The experience of social exclusion in women with a history of suicidal acts: a neuroimaging study. *Sci. Rep.* **7**, 89 (2017).

52. Olvet, D. M. *et al.* A diffusion tensor imaging study of suicide attempters. *J. Psychiatr. Res.* **51**, 60–67 (2014).

53. Oquendo, M. A. *et al.* Positron emission tomography of regional brain metabolic responses to a serotonergic challenge and lethality of suicide attempts in major depression. *Arch. Gen. Psychiatry* **60**, 14–22 (2003).

54. Osuch, E., Ford, K., Wrath, A., Bartha, R. & Neufeld, R. Functional MRI of pain application in youth who engaged in repetitive non-suicidal self-injury vs. psychiatric controls. *Psychiatry Res. - Neuroimaging* **223**, 104–112 (2014).

55. Pan, L. A. *et al.* Dissociable patterns of neural activity during response inhibition in depressed adolescents with and without suicidal behavior. *J. Am. Acad. Child Adolesc. Psychiatry* **50**, 602–611 (2011).

56. Pan, L. A., Ramos, L., Segreti, A. M., Brent, D. A. & Phillips, M. L. Right superior temporal gyrus volume in adolescents with a history of suicide attempt. *Br. J. Psychiatry* **206**, 339–340 (2015).

57. Pan, L. A. *et al.* Differential patterns of activity and functional connectivity in emotion processing neural circuitry to angry and happy faces in adolescents with and without suicide attempt. *Psychol. Med.* **43**, 2129–2142 (2013).

58. Pan, L. *et al.* Preserved hippocampal function during learning in the context of risk in adolescent suicide attempt. *Psychiatry Res. - Neuroimaging* **211**, 112–118 (2013).

59. Peng, H. *et al.* Increased suicide attempts in young depressed patients with abnormal temporal-parietal-limbic gray matter volume. *J. Affect. Disord.* **165**, 69–73 (2014).

60. Plener, P. L., Bubalo, N., Fladung, A. K., Ludolph, A. G. & Lulé, D. Prone to excitement: Adolescent females with non-suicidal self-injury (NSSI) show altered cortical pattern to emotional and NSS-related material. *Psychiatry Res. - Neuroimaging* **203**, 146–152 (2012).

61. Potvin, S., Tikàsz, A., Richard-Devantoy, S., Lungu, O. & Dumais, A. History of suicide attempt is associated with reduced medial prefrontal cortex activity during emotional decision-making among men with schizophrenia: An exploratory fMRI study. *Schizophr. Res. Treatment* 1–8 (2018).

62. Quevedo, K., Martin, J., Scott, H., Smyda, G. & Pfeifer, J. H. The neurobiology of self-knowledge in depressed and self-injurious youth. *Psychiatry Res. - Neuroimaging* **254**, 145–155 (2016).

63. Reitz, S. *et al.* Incision and stress regulation in borderline personality disorder: Neurobiological mechanisms of self-injurious behaviour. *Br. J. Psychiatry* **207**, 165–172 (2015).

64. Richard-Devantoy, S., Ding, Y., Lepage, M., Turecki, G. & Jollant, F. Cognitive inhibition in depression and suicidal behavior: a neuroimaging study. *Psychol. Med.* **46**, 933–944 (2016).

65. Rizk, M. M. *et al.* Gray matter volumetric study of major depression and suicidal behavior. *Psychiatry Res. - Neuroimaging* **283**, 16–23 (2019).

66. Rüsch, N. *et al.* Inferior frontal white matter volume and suicidality in schizophrenia. *Psychiatry Res. - Neuroimaging* **164**, 206–214 (2008).

67. Schmahl, C. *et al.* Neural Correlates of Antinociception in Borderline Personality Disorder. *Arch. Gen. Psychiatry* **63**, 659–667 (2006).

68. Schreiner, M. W. *et al.* Multi-modal neuroimaging of adolescents with non-suicidal self-injury: Amygdala functional connectivity. *J. Affect. Disord.* **221**, 47–55 (2017).

69. Segreti, A. M., Chase, H. W., Just, M., Brent, D. & Pan, L. Cortical thickness and volume reductions in young adults with current suicidal ideation. *J. Affect. Disord.* **245**, 126–129 (2019).

70. Taylor, W. D. *et al.* Widespread white matter but focal gray matter alterations in depressed individuals with thoughts of death. *Prog. Neuro-Psychopharmacology Biol. Psychiatry* **62**, 22–28 (2015).

71. van Heeringen, K., Wu, G. R., Vervaet, M., Vanderhasselt, M. A. & Baeken, C. Decreased resting state metabolic activity in frontopolar and parietal brain regions is associated with suicide plans in depressed individuals. *J. Psychiatr. Res.* **84**, 243–248 (2017).

72. Vanyukov, P. M. *et al.* Paralimbic and lateral prefrontal encoding of reward value during intertemporal choice in attempted suicide. *Psychol. Med.* **46**, 381–391 (2016).

73. Wallace, A. R. Neurocircuitry of suicidal behavior in adolescents and young adults with bipolar and major depressive disorder. *Yale Medicine Thesis Digital Library* (2015).

74. Wei, S. *et al.* Amygdala functional connectivity in female patients with major depressive disorder with and without suicidal ideation. *Ann. Gen. Psychiatry* **17**, 1–7 (2018).

75. Willeumier, K., Taylor, D. V & Amen, D. G. Decreased cerebral blood flow in the limbic and prefrontal cortex using SPECT imaging in a cohort of completed suicides. *Transl. Psychiatry* **1**, e28 (2011).

76. Zhang, H. *et al.* Opposite effective connectivity in the posterior cingulate and medial prefrontal cortex between first-episode schizophrenic patients with suicide risk and healthy controls. *PLoS One* **8**, 1–8 (2013).

77. Zhang, S. *et al.* Association between abnormal default mode network activity and suicidality in depressed adolescents. *BMC Psychiatry* **16**, 337 (2016).

**Table S1. Description of Included Studies.**

| **Paper** | **Imaging Techniques** | **SITB Group N** | **Control Group N** | **Control Type** | **Medication** | **Sample Age** | **Tasks** | **Type of Tasks** | **SITBs Type** | **Number of Contrasts** | **Source of Coordinates** |
| --- | --- | --- | --- | --- | --- | --- | --- | --- | --- | --- | --- |
| Aguilar et al., 2008 | MRI | 13 | 24 | Psychiatric | All Medicated | Adults Only | NA | NA | Suicide Attempt | 2 | Table 2 |
| Ai et al., 2018 | fMRI | 18 | 26 | Healthy | Some Medicated | Adults Only | Emotional Processing | Affective | Suicide Attempt | 2 | Tables 2A & 4 |
|  |  |  |  |  |  |  | Tower of London | Cognitive |  | 1 |  |
|  |  | 18 | 31 | Self-Injurious |  |  | Emotional Processing | Affective |  | 2 |  |
|  |  | 18 | 85 | Self-Injurious + Psychiatric (combined) |  |  |  |  |  | 2 |  |
|  |  | 18 | 111 | Self-Injurious + Psychiatric + Healthy (combined) |  |  | Tower of London | Cognitive |  | 1 |  |
| Alarcon et al., 2019 | fMRI | 82 | 38 | Healthy | Some Medicated | Adolescents Only | Emotional Self-Other Morph-Query (ESOM-Q) Task | Affective | All Suicidal Thoughts and Behaviors | 9 | Table 2 & Table S4 |
| Ambrosi et al., 2018 | fMRI | 123 | 149 | Self-Injurious + Healthy (combined) | Some Medicated | Adults Only | NA | NA | Suicide Attempt | 8 | Table 1 |
| Amen et al., 2009 | SPECT | 9 | 12 | Healthy | Some Medicated | Adults Only | Connor's Continuous Performance Test | Cognitive | Suicide Death | 15 | Table 2 |
|  |  | 12 | 12 | Healthy |  |  |  |  |  | 25 | Table 3 |
|  |  | 9 | 12 | Psychiatric |  |  |  |  |  | 6 | Table 6 |
|  |  | 12 | 12 | Psychiatric |  |  |  |  |  | 15 | Table 7 |
| Audenaert et al., 2002 | SPECT | 20 | 20 | Healthy | Some Medicated | Adults Only | Controlled Oral Word Association Test | Cognitive | Suicide Attempt | 16 | Figures 1-3 |
| Beauchaine et al., 2018 | MRI | 20 | 20 | Healthy | None Medicated | Adolescents and Adults | NA | NA | Self-Harm (Regardless of Intent) | 3 | Table 2 |
| Benedetti et al., 2011 | MRI | 19 | 38 | Psychiatric | Some Medicated | Adults Only | NA | NA | Suicide Attempt | 40 | Table 2 |
| Benedetti et al., 2014 | MRI | 32 | 104 | Psychiatric | Some Medicated | Adults Only | NA | NA | Suicide Attempt | 1 | Figure 3 |
| Besteher et al., 2016 | MRI | 14 | 23 | Psychiatric | All Medicated | Adults Only | NA | NA | Suicide Attempt | 1 | Table 3 |
| Bijttebier et al., 2015 | DTI | 13 | 15 | Psychiatric | Some Medicated | Adults Only | NA | NA | Suicide Attempt | 4 | Table 2 |
|  |  | 13 | 17 | Healthy |  |  |  |  |  | 4 |  |
| Brown et al., 2017 | fMRI | 13 | 32 | Healthy | Some Medicated | Adults Only | Cyberball Game | Affective | NSSI | 1 | Table 2 |
|  |  | 14 | 32 | Healthy |  |  |  |  |  | 4 |  |

**Table S1 - continued**

| **Paper** | **Imaging Techniques** | **SITB Group N** | **Control Group N** | **Control Type** | **Medication** | **Sample Age** | **Tasks** | **Type of Tasks** | **SITBs Type** | **Number of Contrasts** | **Source of Coordinates** |
| --- | --- | --- | --- | --- | --- | --- | --- | --- | --- | --- | --- |
| Cao et al., 2015 | fMRI | 19 | 20 | Healthy | Unclear | Adults Only | NA | NA | Suicide Attempt | 14 | Table 2 |
| Cao et al., 2016 | fMRI | 35 | 18 | Psychiatric | Some Medicated | Adolescents and Adults | NA | NA | Suicide Attempt | 6 | Table 2 |
|  |  | 35 | 47 | Healthy |  |  |  |  |  | 4 |  |
| Chase et al., 2017 | fMRI | 34 | 40 | Healthy | All Medicated | Adults Only | NA | NA | Suicide Ideation | 2 | Figure 2 |
|  |  | 18 | 16 | Self-Injurious |  |  |  |  | Suicide Attempt | 2 |  |
| Chen et al., 2015 | MRI | 17 | 47 | Psychiatric + Healthy (combined) | None Medicated | Adults Only | NA | NA | Suicide Attempt | 2 | Table 2 |
| Davis et al., 2014 | fMRI | 21 | 27 | Healthy | Unclear | Adults Only | Negative Emotional Reactivity and Regulation Task | Affective | Self-Harm (Regardless of Intent) | 6 | Table 5 |
| Ding et al., 2015 | MRI | 67 | 82 | Psychiatric | Some Medicated | Adults Only | NA | NA | Suicide Attempt | 1 | Page 3 |
| Fan et al., 2013 | fMRI | 27 | 9 | Psychiatric | Some Medicated | Adults Only | NA | NA | Suicide Attempt | 2 | Table 2 |
|  |  | 27 | 57 | Healthy |  |  |  |  |  | 5 |  |
| Fan et al., 2019 | DTI | 21 | 25 | Psychiatric | Some Medicated | Adolescents and Adults | NA | NA | Suicide Attempt | 2 | Pages 1092-1093 |
|  | MRI |  |  |  |  |  |  |  |  | 6 |  |
|  | DTI | 19 | 18 | Psychiatric |  |  |  |  |  | 2 |  |
|  | MRI |  |  |  |  |  |  |  |  | 3 |  |
| Fradkin et al., 2016 | MRI | 29 | 29 | Healthy | Some Medicated | Adolescents and Adults | NA | NA | Suicide Attempt | 4 | Table 2 |
| Garrion et al., 2008 | fMRI | 7 | 9 | Psychiatric | None Medicated | Adolescents Only | Go/No-Go Task | Cognitive | Self-Harm (Regardless of Intent) | 4 | Table 5 |
| Groschwitz et al., 2016 | fMRI | 14 | 29 | Psychiatric + Healthy (combined) | Some Medicated | Adolescents Only | Cyberball Game | Affective | NSSI | 3 | Table 2 |
|  |  | 14 | 14 | Psychiatric |  |  |  |  |  | 3 | Table 3 |
| Harenski et al., 2018 | MRI | 19 | 46 | Psychiatric + Healthy (combined) | Unclear | Adults Only | NA | NA | Suicide Attempt | 39 | Table 3 |

**Table S1 – continued**

| **Paper** | **Imaging Techniques** | **SITB Group N** | **Control Group N** | **Control Type** | **Medication** | **Sample Age** | **Tasks** | **Type of Tasks** | **SITBs Type** | **Number of Contrasts** | **Source of Coordinates** |
| --- | --- | --- | --- | --- | --- | --- | --- | --- | --- | --- | --- |
| Hwang et al., 2010 | MRI | 27 | 43 | Psychiatric | Unclear | Elderly Only | NA | NA | Suicide Attempt | 54 | Tables 3 & 4 |
| Jia et al., 2010 | DTI | 16 | 36 | Psychiatric | None Medicated | Adults Only | NA | NA | Suicide Attempt | 2 | Table 2 |
|  |  | 16 | 52 | Healthy |  |  |  |  |  | 2 |  |
| Johnston et al., 2017 | fMRI | 26 | 42 | Psychiatric | Some Medicated | Adolescents and Adults | Emotion Face Processing Task | Affective | Suicide Attempt | 4 | Table 2 |
|  | MRI |  |  |  |  |  | NA | NA |  | 3 |  |
|  | DTI |  |  |  |  |  |  |  |  | 3 |  |
| Jollant et al., 2008 | fMRI | 13 | 14 | Psychiatric | Some Medicated | Adults Only | Emotion Face Viewing Task | Affective | Suicide Attempt | 5 | Table 3 |
|  |  | 13 | 30 | Psychiatric + Healthy (combined) |  |  |  |  |  | 7 |  |
|  |  | 13 | 16 | Healthy |  |  |  |  |  | 2 |  |
| Jollant et al., 2010 | fMRI | 13 | 12 | Psychiatric | Some Medicated | Adults Only | Iowa Gambling Task | Cognitive | Suicide Attempt | 3 | Page 1278 |
| Just et al., 2017 | fMRI | 17 | 17 | Healthy | Unclear | Adults Only | Actively thinking about words related to suicide, negative affect, and positive affect | Affective | Suicide Ideation | 5 | Table 3 |
| Kang et al., 2017 | fMRI | 19 | 19 | Psychiatric | Some Medicated | Adults Only | NA | NA | Suicide Attempt | 3 | Tables 1 & 2 |
|  |  | 19 | 19 | Self-Injurious |  |  |  |  | All Suicidal Thoughts and Behaviors | 1 |  |
| Kim et al., 2017 | fMRI | 14 | 22 | Healthy | Unclear | Adults Only | Viewing pictures of faces, suicidal means, and natural landscapes | Affective | Suicide Attempt | 4 | Figure 2 |
| Kraus et al., 2010 | fMRI | 11 | 10 | Healthy | None Medicated | Adults Only | Script-Driven Imagery of Self-Injurious Behaviors | Affective | Self-Harm (Regardless of Intent) | 2 | Page 46 |
| Lan et al., 2018 | fMRI | 26 | 15 | Psychiatric | None Medicated | Adults Only | NA | NA | Suicide Ideation | 1 | Page 4 |
| Lee et al., 2015 | fMRI | 14 | 31 | Psychiatric + Healthy (combined) | Unclear | Adults Only | Go/No-Go Task | Cognitive | Self-Harm (Regardless of Intent) | 6 | Table 3 |
| Lee, Kim, Gwak et al., 2016 | MRI | 19 | 19 | Psychiatric | All Medicated | Adults Only | NA | NA | Suicide Attempt | 2 | Table 2 |
| Lee, Kim, Oh et al., 2016 | DTI | 15 | 41 | Psychiatric | Some Medicated | Adults Only | NA | NA | Suicide Attempt | 1 | Page 25 |
| Leyton et al., 2006 | PET | 10 | 16 | Healthy | None Medicated | Adults Only | NA | NA | Suicide Attempt | 5 | Page 222 |

**Table S1 - continued**

| **Paper** | **Imaging Techniques** | **SITB Group N** | **Control Group N** | **Control Type** | **Medication** | **Sample Age** | **Tasks** | **Type of Tasks** | **SITBs Type** | **Number of Contrasts** | **Source of Coordinates** |
| --- | --- | --- | --- | --- | --- | --- | --- | --- | --- | --- | --- |
| Li et al., 2018 | fMRI | 28 | 20 | Psychiatric | None Medicated | Adults Only | NA | NA | Suicide Ideation | 4 | Table 2 |
| Lippard et al., 2019 | DTI | 17 | 29 | Psychiatric | Some Medicated | Adolescents and Adults | NA | NA | Suicide Attempt | 8 | Page 1142 |
|  | MRI |  |  |  |  |  |  |  |  | 4 |  |
| Long et al., 2018 | DTI | 18 | 45 | Psychiatric | Some Medicated | Adults Only | NA | NA | Suicide Ideation | 2 | Figures 2 & 3 |
| Mahon et al., 2012 | DTI | 14 | 15 | Psychiatric | Some Medicated | Adults Only | NA | NA | Suicide Attempt | 2 | Figure 1 |
| Marchand et al., 2012 | fMRI | 6 | 16 | Psychiatric | None Medicated | Adults Only | Self-Paced Button Pressing Task | Other | Self-Harm (Regardless of Intent) | 14 | Tables 3, 4, & 5 |
|  |  | 13 | 9 | Psychiatric |  |  |  |  | Suicide Ideation | 7 | Tables 2 & 4 |
| Matthews et al., 2012 | fMRI | 13 | 13 | Psychiatric | Some Medicated | Adults Only | Stop Task | Cognitive | Suicide Ideation | 12 | Table 3 |
| Miller et al., 2018 | fMRI | 14 | 32 | Healthy | None Medicated | Adolescents and Adults | Viewing positive, negative, or neutral images | Affective | Suicide Ideation | 6 | Table 2 |
| Minzenberg et al., 2015 (Journal of Affective Disorders) | fMRI | 16 | 14 | Psychiatric | All Medicated | Adults Only | Continuous Performance Task (AX Version) | Cognitive | Suicide Ideation | 3 | Table 2 |
|  |  | 8 | 8 | Self-Injurious |  |  |  |  | All Suicidal Behaviors | 6 |  |

**Table S1 – Continued**

| **Paper** | **Imaging Techniques** | **SITB Group N** | **Control Group N** | **Control Type** | **Medication** | **Sample Age** | **Tasks** | **Type of Tasks** | **SITBs Type** | **Number of Contrasts** | **Source of Coordinates** |
| --- | --- | --- | --- | --- | --- | --- | --- | --- | --- | --- | --- |
| Minzenberg et al., 2015 (Journal of Psychiatric Research) | fMRI | 15 | 17 | Psychiatric | Some Medicated | Adults Only | Continuous Performance Task (AX Version) | Cognitive | Suicide Ideation | 10 | Table 2 |
|  |  | 8 | 24 | Self-Injurious + Psychiatric (combined) |  |  |  |  | All Suicidal Thoughts and Behaviors | 20 |  |
| Minzenberg et al., 2016 | fMRI | 16 | 14 | Psychiatric | All Medicated | Adults Only | Continuous Performance Task (AX Version) | Cognitive | Suicide Ideation | 24 | Table 2 |
|  |  |  |  |  |  |  |  |  | Suicide Ideation & Plan | 10 |  |
|  |  | 8 | 8 | Self-Injurious |  |  |  |  | All Suicidal Behaviors | 11 |  |
| Niedtfeld et al., 2010 | fMRI | 20 | 23 | Healthy | None Medicated | Adults Only | Viewing Negative and Neutral Valence Pictures and Receiving Thermal Stimuli^†^ | Affective | Self-Harm (Regardless of Intent) | 32 | Tables 1 & 3 |
|  |  |  |  |  |  |  |  | Affective & Pain |  | 8 |  |
|  |  |  |  |  |  |  |  | Pain |  | 9 |  |
| Niedtfeld et al., 2012 | fMRI | 20 | 23 | Healthy | None Medicated | Adults Only | Viewing Negative and Neutral Valence Pictures and Receiving Thermal Stimuli^†^ | Affective | Self-Harm (Regardless of Intent) | 3 | Tables S2, S3, & S6 |
|  |  |  |  |  |  |  |  | Affective & Pain |  | 10 | Tables S1, S3-6 |
|  |  |  |  |  |  |  |  | Pain |  | 23 | Tables S1-2, & S4-6 |
| Olié et al., 2017 | fMRI | 36 | 69 | Psychiatric + Healthy (combined) | Some Medicated | Adults Only | Cyberball Game | Affective | Suicide Attempt | 2 | Figure 2 |
| Olvet et al., 2014 | DTI | 13 | 39 | Psychiatric | None Medicated | Adults Only | NA | NA | Suicide Attempt | 1 | Table 5 |
| Oquendo et al., 2003 | PET | 16 | 9 | Self-Injurious | None Medicated | Adults Only | Fenfluramine Injection | Other | Suicide Attempt | 9 | Figure 1 |
| Osuch et al., 2014 | fMRI | 13 | 15 | Psychiatric | Some Medicated | Adolescents and Adults | Self-administered and Experimenter-administered cold stimuli | Pain | NSSI | 35 | Tables 2 & 3, Figure 5 |

^†^ The exact task type is dependent on the specific contrasts. Given that there were not sufficient contrasts to analyze pain as a task, when tasks involved both affective and pain components, they were grouped with affective tasks.

**Table S1 - continued**

| **Paper** | **Imaging Techniques** | **SITB Group N** | **Control Group N** | **Control Type** | **Medication** | **Sample Age** | **Tasks** | **Type of Tasks** | **SITBs Type** | **Number of Contrasts** | **Source of Coordinates** |
| --- | --- | --- | --- | --- | --- | --- | --- | --- | --- | --- | --- |
| Pan et al., 2011 | fMRI | 15 | 29 | Psychiatric + Healthy (combined) | Some Medicated | Adolescents Only | Go/No-Go Task | Cognitive | Suicide Attempt | 3 | Table 2 |
| Pan et al., 2015 | MRI | 28 | 31 | Psychiatric | Some Medicated | Adolescents Only | NA | NA | Suicide Attempt | 3 | Table DS2 |
|  |  | 28 | 41 | Healthy |  |  |  |  |  | 13 |  |
| Pan, Hassel et al., 2013 | fMRI | 14 | 15 | Psychiatric | Some Medicated | Adolescents Only | Emotion Face Viewing Task | Affective | Suicide Attempt | 22 | Tables 3-5 |
|  |  | 14 | 15 | Healthy |  |  |  |  |  | 17 |  |
| Pan, Segreti, et al., 2013 | fMRI | 15 | 14 | Psychiatric | Some Medicated | Adolescents Only | Iowa Gambling Task | Cognitive | Suicide Attempt | 6 | Tables 2 & 3 |
|  |  | 15 | 13 | Healthy |  |  |  |  |  | 7 |  |
| Peng et al., 2014 | MRI | 20 | 18 | Psychiatric | Some Medicated | Adults Only | NA | NA | Suicide Attempt | 1 | Table 2 |
|  |  | 20 | 28 | Healthy |  |  |  |  |  | 2 |  |
| Plener et al., 2012 | fMRI | 9 | 9 | Healthy | Some Medicated | Adolescents Only | Viewing Emotional IAPS Pictures and NSSI Pictures | Affective | NSSI | 15 | Table 3 |
| Potvin et al., 2018 | fMRI | 13 | 19 | Psychiatric | Some Medicated | Adults Only | Balloon Analog Risk Task | Cognitive | Suicide Ideation | 3 | Tables 3 & 4 |
|  |  | 13 | 21 | Healthy |  |  |  |  |  | 5 |  |
| Quevedo et al., 2016 | fMRI | 50 | 73 | Psychiatric + Healthy (combined) | Some Medicated | Adolescents Only | Interpersonal Self-Processing task | Affective | NSSI | 10 | Table 2 |
| Reitz et al., 2015 | fMRI | 21 | 17 | Healthy | None Medicated | Adults Only | Montreal Imaging Stress Task and Pain Induction | Affective | NSSI | 6 | Table 2 |
| Richard-Devantoy et al., 2016 | fMRI | 26 | 28 | Healthy | None Medicated | Adults Only | Go/No-Go Task | Cognitive | Suicide Attempt | 1 | Supplement |
|  |  | 26 | 51 | Psychiatric+ Healthy (combined) |  |  |  |  |  | 1 |  |
| Rizk et al., 2018 | MRI | 11 | 80 | Psychiatric + Self-Injurious (combined) | None Medicated | Adults Only | NA | NA | Suicide Attempt | 1 | Page 20 |
| Rüsch et al., 2008 | MRI | 10 | 45 | Psychiatric | Some Medicated | Adults Only | NA | NA | Suicide Attempt | 2 | Page 209 |
| Schmahl et al., 2006 | fMRI | 12 | 12 | Healthy | None Medicated | Adults Only | Thermal Stimuli | Pain | Self-Harm (Regardless of Intent) | 6 | Table 3 |
| Schreiner, 2017 | fMRI | 25 | 20 | Healthy | Some Medicated | Adolescents and Adults | Emotion Face Matching Task | Affective | NSSI | 8 | Table 2 |
|  |  | 24 | 17 | Healthy |  |  |  |  |  | 15 | Tables 3, S1, S2 |

**Table S1 – continued**

| **Paper** | **Imaging Techniques** | **SITB Group N** | **Control Group N** | **Control Type** | **Medication** | **Sample Age** | **Tasks** | **Type of Tasks** | **SITBs Type** | **Number of Contrasts** | **Source of Coordinates** |
| --- | --- | --- | --- | --- | --- | --- | --- | --- | --- | --- | --- |
| Segreti et al., 2018 | MRI | 35 | 43 | Healthy | All Medicated | Adults Only | NA | NA | Suicide Ideation | 3 | Figure 1 |
| Taylor et al., 2015 | DTI | 21 | 53 | Psychiatric | None Medicated | Adults Only | NA | NA | Suicide Ideation | 2 | Table 2 |
|  |  | 21 | 91 | Healthy |  |  |  |  |  | 4 |  |
|  | MRI | 21 | 53 | Psychiatric |  |  |  |  |  | 4 | Figure 1 |
| Van Heeringen et al., 2017 | PET | 17 | 20 | Healthy | All Medicated | Adults Only | NA | NA | Suicide Plan | 4 | Table 2 |
|  |  | 17 | 12 | Psychiatric |  |  |  |  |  | 1 |  |
|  |  | 17 | 11 | Self-Injurious |  |  |  |  | Suicide Ideation | 3 |  |
| Vanyukov et al., 2016 | fMRI | 13 | 35 | Psychiatric + Healthy (combined) | Unclear | Adults and Elderly | Delay Discounting Task | Cognitive | Suicide Attempt | 1 | Figure 2 |
| Wallace 2015 | DTI | 21 | 25 | Psychiatric | Some Medicated | Adolescents and Adults | NA | NA | Suicide Attempt | 3 | Table 4 |
|  |  | 18 | 17 |  |  |  |  |  |  | 1 | Table 5 |
|  |  | 39 | 42 |  |  |  |  |  |  | 6 | Table 3 |
|  |  | 21 | 43 | Healthy |  |  |  |  |  | 3 |  |
|  |  | 18 |  |  |  |  |  |  |  | 1 |  |
|  |  | 39 |  |  |  |  |  |  |  | 6 |  |
| Wei et al., 2018 | fMRI | 15 | 63 | Psychiatric + Healthy (combined) | Unclear | Adults Only | NA | NA | Suicide Ideation | 1 | Page 4 |
| Willeumier et al., 2011 | SPECT | 21 | 36 | Psychiatric | None Medicated | Adolescents and Adults | NA | NA | Suicide Death | 10 | Table 4 |
|  |  | 21 | 27 | Healthy |  |  |  |  |  | 10 | Table 3 |
| Zhang et al., 2013 | fMRI | 14 | 34 | Psychiatric + Healthy (combined) | Some Medicated | Adults Only | N-Back Task | Cognitive | Suicide Risk | 2 | Page 3 |
| Zhang et al., 2016 | fMRI | 35 | 18 | Psychiatric | None Medicated | Adolescents and Adults | NA | NA | Suicide Attempt | 3 | Table 2 |
|  |  | 35 | 47 | Healthy |  |  |  |  |  | 5 |  |
